# Sludge Biochar Amendment and Alfalfa Revegetation Improve Soil Physicochemical Properties and Increase Diversity of Soil Microbes in Soils from a Rare Earth Element Mining Wasteland

**DOI:** 10.3390/ijerph15050965

**Published:** 2018-05-11

**Authors:** Caigui Luo, Yangwu Deng, Kazuyuki Inubushi, Jian Liang, Sipin Zhu, Zhenya Wei, Xiaobin Guo, Xianping Luo

**Affiliations:** 1 Faculty of Resources and Environmental Engineering, Jiangxi University of Science and Technology, Ganzhou 341000, China; andk24@163.com (C.L.); ljreee@163.com (J.L.); zhusipin926@163.com (S.Z.); m15607077016@163.com (Z.W.); 2 National Engineering Research Center for Ionic Rare Earth, Ganzhou 341000, China; guoxb1964@163.com; 3 Jiangxi Key Laboratory of Mining & Metallurgy Environmental Pollution Control, Ganzhou 341000, China; 4 Graduate School of Horticulture, Chiba University, Matsudo, Chiba 2718510, Japan; inubushi@faculty.chiba-u.jp

**Keywords:** sludge biochar amendment, alfalfa revegetation, bacterial and fungal communities, ion-adsorption rare earth mining wasteland, soil physicochemical properties

## Abstract

Long-term unregulated mining of ion-adsorption clays (IAC) in China has resulted in severe ecological destruction and created large areas of wasteland in dire need of rehabilitation. Soil amendment and revegetation are two important means of rehabilitation of IAC mining wasteland. In this study, we used sludge biochar prepared by pyrolysis of municipal sewage sludge as a soil ameliorant, selected alfalfa as a revegetation plant, and conducted pot trials in a climate-controlled chamber. We investigated the effects of alfalfa revegetation, sludge biochar amendment, and their combined amendment on soil physicochemical properties in soil from an IAC mining wasteland as well as the impact of sludge biochar on plant growth. At the same time, we also assessed the impacts of these amendments on the soil microbial community by means of the Illumina Miseq sequences method. Results showed that alfalfa revegetation and sludge biochar both improved soil physicochemical properties and microbial community structure. When alfalfa revegetation and sludge biochar amendment were combined, we detected additive effects on the improvement of soil physicochemical properties as well as increases in the richness and diversity of bacterial and fungal communities. Redundancy analyses suggested that alfalfa revegetation and sludge biochar amendment significantly affected soil microbial community structure. Critical environmental factors consisted of soil available K, pH, organic matter, carbon–nitrogen ratio, bulk density, and total porosity. Sludge biochar amendment significantly promoted the growth of alfalfa and changed its root morphology. Combining alfalfa the revegetation with sludge biochar amendment may serve to not only achieve the revegetation of IAC mining wasteland, but also address the challenge of municipal sludge disposal by making the waste profitable.

## 1. Introduction

Ion-adsorption clay (IAC) contains rare earth elements (REEs). IAC mines are widely distributed throughout several adjacent provinces of southern China, including Jiangxi, Guangdong, Fujian, Guangxi, Hunan, Yunnan, Zhejiang provinces [1]. In the early days of mining, IAC was extracted via tank or heap leaching [2]. These methods cause serious ecological destruction in IAC mining wastelands [3], including loss of vegetation, pollution of water and soil, and geological disasters (e.g., landslides). As a result, the soil in IAC mining wastelands has a loose texture, poor aggregation, low water-holding capacity and fertility, and decreased microbial diversity, all of which makes it hard for plants to colonize these soils. Rehabilitation is urgently needed for IAC mining wastelands and two potential measures are soil amendment and revegetation.

A key step for soil amendment is the selection of an appropriate soil ameliorant. Biochar, which is prepared by slow pyrolysis of biomass under oxygen-limited conditions, has been a focus of research on soil amendments [4,5,6,7,8,9,10]. The characteristics of biochar determine how it could improve soil properties. Its porous structure can increase soil porosity [11,12], reduce soil bulk density [13], and provide a habitat for microorganisms [14]. Furthermore, the huge surface area [15] and abundant functional groups (e.g., carboxyls and phenolic hydroxyls) [16] could enhance soil cation exchange capacity (CEC), increase water-holding capacity and decrease fertilizer leaching [17]. The aromatic hydrocarbon structure contributes to the long-term retention of biochar in soil [18]. Biochar can be prepared from a wide range of raw materials, such as agricultural waste [5], animal manure [19], and municipal sludge [20]. It has been proposed that municipal sewage sludge may be an important raw material for biochar preparation because it is rich in mineral nutrients (N, P and K) and organic matter [21].

China’s yield of municipal sludge has been growing rapidly, with an average increase of more than 10% each year from 2008 to 2014 and an estimated 34 million tons produced in 2015 [22]. More than 20% of municipal sludge, which contains pathogens, heavy metals and other pollutants, remains stacked on land on the outskirts of cities [23] where it could threaten human health by entering food or water supplies [24]. Currently, the disposal of municipal sludge is an urgent problem for the government and society. Traditional methods of municipal sludge disposal include agricultural applications, incineration and landfill disposal, all of which have drawbacks, including the fact that these methods require a lot of land resources and result in air pollution, and heavy-metal pollution in soil and water. Pyrolysis is an effective way to dispose of sludge, which is transformed into sludge biochar and used as a soil-amending resource. In addition, sludge biochar can prevent the leaching of heavy metals in raw sewage sludge [25]. Many studies have reported the application of sludge biochar in the remediation of different kinds of sites. Méndez et al. [26] evaluated the effects of sludge biochar derived from sewage sludge on heavy metals solubility and bioavailability in a Mediterranean agricultural soil; the result showed that the risk of leaching of Cu, Ni and Zn were lower in the soil treated with sludge biochar, which also reduced plant availability of Ni Zn, Cd and Pb when compared with soil treated with raw sewage sludge. Sardar Khan. et al. [18] investigated the impact of sludge biochar upon rice (*Oryza sativa* L.) yield, metal bioaccumulation and greenhouse gas emissions from acidic paddy soil, and concluded that sludge biochar increased soil pH, total nitrogen, soil organic carbon and available nutrients and decreased bioavailable As, Cr, Co, Ni, and Pb in soil as well as significantly (*p* ≤ 0.01) increasing shoot biomass, grain yield and the bioaccumulation of phosphorus and sodium. Méndez et al. [27] assessed the influence of sewage sludge and sewage sludge biochar on peat properties as growing media and on lettuce (*Lactuca sativa*) growth, and they confirmed that sewage sludge transformation into biochar proved to be a sustainable waste management approach in order to promote their future use as growing media components. To our knowledge, little is known about the effect of the use of sludge biochar on the soil properties of IAC mining wastelands.

Revegetation is an important measure used for rehabilitation and a critical step in the use of this measure is the best choice of revegetation plants for a particular site. Alfalfa is an attractive revegetation plant, an important gramineous forage legume, because of its adaptability to climates and soil environments, rapid growth, high yield, and ability to fix nitrogen [28,29]. Alfalfa is widely used in the remediation of heavy metals, oil, and other contaminated soils as well as to ameliorate the effects of degraded soil [30,31]. Alfalfa revegetation has not been tested for soil restoration of IAC mining wastelands.

Microorganisms in soil play a critical role in cycling materials. The sensitivity of the response of a microbial community, specifically bacterial and fungal communities, reflects a change in soil quality [32,33]. For instance, a significant alteration of microbial community in IAC mining wasteland was demonstrated in our previous study [34] and some other reports [35,36,37]. In this study, we used sludge biochar as a soil ameliorant and alfalfa as the revegetation plant and conducted pot trials in a climate-controlled chamber, and we test the hypothesis that sludge biochar amendment and alfalfa plantation can ameliorate physicochemical properties and increase the microbial diversity of soil from IAC mining wastelands. Our objectives were to determine the effects of alfalfa revegetation and municipal sludge biochar amendment, and both in combination, on the physicochemical properties and microbial communities of soil from IAC mining wastelands. We aimed to not only explore a new way to utilize municipal sludge as a resource, but also to test a new rehabilitation method for IAC mining wastelands.

## 2. Materials and Methods

### 2.1. Experimental Soil, Preparation of Sludge Biochar, and Revegetation Plant

The experimental soil was collected from the top layer (0–20 cm) of the stockpiled soil (an Entisol, specifically an Udarent) after mining in the Chakeng IAC mining wasteland (24°56′17” N, 115°03′22” E), located in Dingnan county, Ganzhou city, Jiangxi province, China. The area has a subtropical humid monsoon climate, with an annual average temperature of 19 °C, an annual rainfall of 1550 mm, and is located in hilly terrain.

The soil was air-dried for 2 day at room temperature, then passed through a 2 mm mesh sieve and kept at 4 °C for subsequent trials. The municipal sludge was collected from the dewatering room in the Baitashang wastewater treatment plant (25°89′91” N, 114°94′05” E) in Ganzhou. The sludge biochar was prepared from air-dried municipal sludge through pyrolysis in a muffle furnace (FO810C, Yamato Scientific, Chongqing, China) in 500 °C for 2 h under a continuous flow of nitrogen. The basic physicochemical properties of the experimental soil and sludge biochar are shown in Table 1. Alfalfa (*Medicago sativa*) was selected as revegetation plant in this study. Alfalfa seeds (purity: >98%, germinability: >85%, from Lantian seed industry Co Ltd., Heze, China) were surface-sterilized with 2% (*volum*/*volum*) hydrogen peroxide for 5 min, then thoroughly rinsed three times with de-ionized water before used in the pot experiment [30].

### 2.2. Trial Set-Up and Sampling

We designed four treatments (Table 2): (1) control soil (CK); (2) soil planted with alfalfa (G); (3) soil amended with 5% (*weight*/*weight*) sludge biochar (SBC); and (4) soil amended with 5% (*w*/*w*) sludge biochar and planted with alfalfa (SBCG). Each treatment was replicated three times. Plastic pots (20.5 cm diameter, 19.5 cm tall) filled with soil or a mixture of soil and sludge biochar, 5 kg per pot, were placed into a climate-controlled chamber for a two-week stabilization. After that, the preconditioned alfalfa seeds, 5 g per pot, were sown for G and SBCG treatments. All pots were then incubated further in the climate chamber (25 °C day, 20 °C night, 12/12 h). Alfalfa sprouted in 2 day. During both the stabilization and incubation period, the position of each pot was adjusted randomly every 2 day to minimize location effects.

When the alfalfa plants reached maturity (about 90 day), a soil sample was taken from each pot and divided into two sub-samples. One sub-sample (5 g fresh soil) was immediately stored in a refrigerator at −80 °C until it was used for DNA extraction. The other sub-sample was air-dried, passed successively through 2 mm and 0.25 mm mesh sieves, and stored at 4 °C for physicochemical analysis (about 1000 g air-dried soil). The plants were harvested from each pot in the G and SBCG treatments and cleaned with tap water and de-ionized water successively for 2–3 min and kept for biomass measurement and root morphology analysis (refer to Section 2.5).

### 2.3. Analysis of Soil Physicochemical Properties

Analysis of soil physicochemical properties was performed according to the standard methods. Specific gravity, bulk density, and total porosity were measured according to [38]. The water-holding capacity of the soil was determined using a previously published method [36]. The determination of the soil’s pH level was measured using a pH-meter (STARTER 3100; OHAUS Instruments, Shanghai, China) with a soil–liquid ratio of 1:2.5. Organic matter was determined by potassium dichromate (K_2_Cr_2_O_7_) oxidation followed by ammonium ferrous sulfate titration. Total nitrogen was measured by high concentration H_2_SO_4_ digestion followed by semi-micro Kjeldahl distillation. Available nitrogen was determined by the alkali solution diffusion method. Determination of available phosphorus was undertaken by the HCl-NH_4_F extraction-molybdenum antimony colorimetric method. Available potassium was analyzed by CH_3_COONH_4_ extraction followed by atomic absorption spectrophotometry.

### 2.4. Analysis of Soil Microbiota

#### 2.4.1. Extraction of Soil DNA

A 200 mg sub-sample of fresh soil was taken from every sample to extract total community genomic DNA using an E.Z.N.A^TM^ Mag-Bind Soil DNA Kit (OMEGA Bio-tek, Norcross, GA, USA), according to the manufacturer’s instructions.

#### 2.4.2. Gene Amplification by Polymerase Chain Reaction (PCR) and Illumina Sequencing

The V4 hyper-variable region of the *16S rRNA* gene and internal transcribed spacer region (ITS) were selected as the bacterial and fungal target segments, respectively. The general primers of bacterial *16S rRNA* gene were 515F (CCC TAC ACG ACG CTC TTC CGA TCTN (barcode) GTG CCA GCM GCC GCG GTAA) and 805R [39] (GAC TGG AGT TCC TTG GCA CCC GAG AAT TCC AGG ACT ACH VGG GTA TCT AAT CC). The general primers of fungal ITS rRNA were ITS-F (CCC TAC ACG ACG CTC TTC CGA TCTN (barcode) CTT GGT CAT TTA GAG GAA GTAA) and ITS-R (GTG ACT GGA GTT CCT TGG CAC CCG AGA ATT CCA GCT GCG TTC TTC ATC GAT GC). All gene amplification was conducted in two rounds with Illumina bridge polymerase chain reaction (PCR)-compatible primers in the second round PCR. The PCR reaction system contained 15 μL 2 × Taq master Mix; 1 μL Bar-PCR primer F (10 μM) and primer F (10 μM) for the first and second round, respectively; 1 μL Primer R (10 μM); 10 ng and 20 ng genomic DNA for the first and second round, respectively; H_2_O (added to 30 μL). The two rounds of PCR were conducted in a T100^TM^ Thermal Cycler (Bio-Rad Laboratories, Inc., Hercules, CA, USA). The first round of PCR consisted of an initial denaturation step at 94 °C for 3 min, followed by five cycles of 94 °C for 30 s, 45 °C for 20 s, and 65 °C for 20 s, followed by another 20 cycles of 94 °C for 20 s, 55 °C for 20 s, and 72 °C for 30 s, and a final extension step at 72 °C for 5 min. The second round of PCR consisted of a denaturation step at 95 °C for 30 s, followed by five cycles of 95 °C for 15 s, 55 °C for 15 s, and 72 °C for 30 s, and a final extension step at 72 °C for 5 min. After two rounds of PCR, the size of the PCR products was confirmed by an agarose gel electrophoresis test. The accurate concentrations of purified products were determined using a Qubit 2.0 Fluorometer (Thermo Fisher Scientific, Waltham, MA, USA) and the amplicons from each reaction mixture were then pooled in equi-molar ratios based on their concentration. The treated samples were sent to Sangon BioTech (Shanghai, China) to sequence on the Illumina MiSeq system (Illumina, San Diego, CA, USA), according to the manufacturer’s instructions.

#### 2.4.3. High-Throughput Sequencing Data Processing

Data from Illumina sequencing was processed and optimized as follows: (1) according to the overlap relationship between paired-end (PE) reads, pair reads were merged into a sequence using the PEAR 0.9.6 software (main parameter: −x 0.1) (Scientific Computing Group, Heidelberg, Germany), and the individual FASTA and QUAL files were generated from the processing of FASTQ files and then analyzed by standard methods; (2) ambiguous sequences were deleted and those with a maximum homo-polymer length of 6 bp were allowed [40]; (3) all identical sequences were merged into one and we recorded the frequency of each sequence to reduce the data calculations; (4) we referred to a customized reference database to align sequences [41]; (5) the completeness of the index and adaptor was checked, and all incomplete sequences were removed; (6) the pre-cluster tool was used to remove noise and the chimera UCHIME algorithm was used to detect chimera sequences; (7) to standardize the sequencing depth of all samples, after quality control, 39,000 bacterial and 30,000 fungal sequences were randomly selected from each sample for the subsequent analyses.

The effective sequences of each sample were submitted to the Ribosomal Database Project (RDP) Classifier (http://rdp.cme.msu.edu/) to identify bacterial and fungal sequences [42]. Mothur [43] was used to calculate microbial richness (ACE and Chao1), diversity (Shannon and Simpson), and coverage.

### 2.5. Analysis of Plant Growth and Their Root Morphology

Plant height was measured with a ruler. The plants from the G and SBCG treatments were oven-dried at 105 °C to determine their biomass as dry weight (shoot, root, and total). We used a root-scanning system (Wanshen Detection Technology Corp., Hangzhou, China) to evaluate the morphology of alfalfa root, including total root length (TRL), root surface area (RSA), root volume (RV), root average diameter (RAD), root tip number (RTN), and root fork number (RFN) [44].

### 2.6. Statistical Analysis

Soil physicochemical properties, richness (ACE and Chao1), and diversity (Shannon and Simpson) of bacterial and fungal communities (phylum and genus) among four treatments as well as the data of plant growth and their root morphology in G and SBCG treatments were compared by one-way analysis of variance (ANOVA), followed by the Duncan’s multiple range test (DMRT), with significance set to *p* < 0.05 level [45]. All analyses were conducted in SPSS 17.0 (SPSS Inc., Chicago, IL, USA). All plots were created using Origin 8.5 Pro software (OriginLab Corporation, Northampton MA, USA). The principal co-ordinate analyses (PCoA) were conducted with R software to determine the similarity and differences of microbial community among four treatments at the genus level.

The multiple relationships between bacterial and fungal community structure, and soil physicochemical properties were carried out in Canoco 4.5 (Biomeris, Wageningen, The Netherlands). De-trended corresponded analyses (DCA) [46], with bacterial and fungal composition data were conducted and the lengths of the first DCA ordination axis were 0.109 and 0.325, which indicated that redundancy analysis (RDA), based on a linear model, should be applied to ordinate the soil bacterial and fungal community structures with physicochemical properties, respectively. The manual selection mode of Monte Carlo permutation test with 499 unrestricted permutations was used to confirm the key environmental factors that significantly influenced the microbial community [47].

## 3. Results and Discussion

### 3.1. Response of Soil Physicochemical Properties to Alfalfa Revegetation and Sludge Biochar Amendment

#### 3.1.1. Effects on Soil Physical Properties

**Figure 1:**
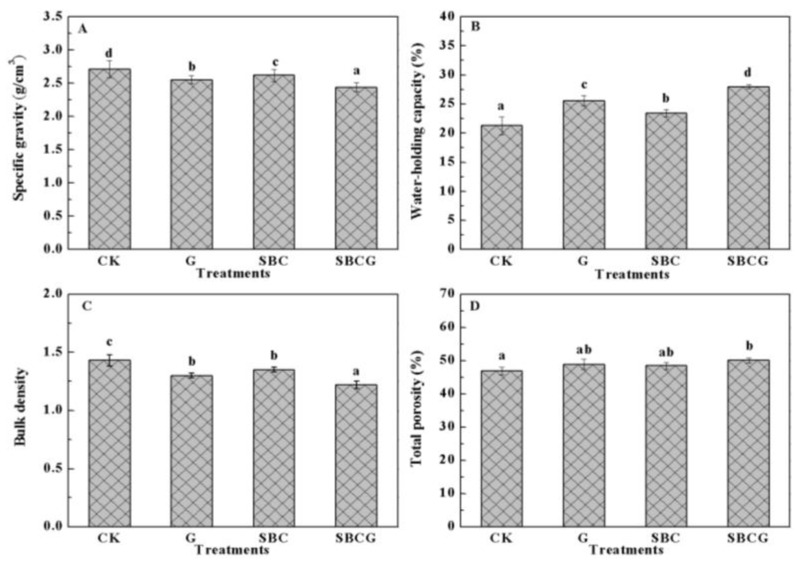
The physical properties ((**A**–**D**) stand for specific gravity, water-holding capacity, bulk density, total porosity, respectively) of ion-adsorption clay (IAC) mining wasteland soil in the un-amended control (CK), alfalfa revegetation (G), sludge biochar amendment (SBC) and combined amendment (SBCG) treatments. Different characters, a, b, c, and d, were used to indicate significant differences at *p* < 0.05. Vertical bars indicate standard deviation.

The soil physical properties of four treatments are shown in Figure 1. There were significant differences among four treatments. The specific gravity values of G (2.55 ± 0.02), SBC (2.62 ± 0.02), and SBCG (2.44 ± 0.02) treatments were all significantly lower than that of the CK treatment (2.70 ± 0.03) (*p* < 0.05). A similar trend was also observed in the bulk density of four treatments, with those of G, SBC, and SBCG significantly lower than that of CK (*p* < 0.05) by 9.1%, 5.6% and 14.7%, respectively. The lower levels of specific gravity and bulk density in treated soils (G, SBC, and SBCG) suggested that sludge biochar application and alfalfa revegetation both significantly reduced soil compaction and enhanced soil porosity and ventilation, which are attributed to the porous structure of the sludge biochar [18] and the strong root system of alfalfa [48,49]. Total porosity only showed significant differences between CK and SBCG (*p* < 0.05). The soil water-holding capacity values of G, SBC, and SBCG were all significantly higher, by 20.2%, 10.2%, and 31.4% (*p* < 0.05), respectively, than that of CK (21.59 ± 1.52%). A previous study [37] showed that long-term exploitation of rare earth resources had destroyed soil structure (poor porosity and ventilation) in an IAC mining wasteland. The significant increases of total porosity and water-holding capacity in SBCG treatment suggested that the combination of alfalfa revegetation and sludge biochar amendment not only improved soil porosity but also helped maintain water content. Many previous studies have demonstrated that the application of biochar can improve the soil physical properties [50], such as soil aggregation [51], water-retention capacity [52,53], pore-size distribution [54], and bulk density [55]. The application of sludge biochar, by means of providing nutrients (N, P, K) [56], can benefit the growth of alfalfa, which in turn helps ameliorate the soil’s physical properties [57]. An additive effect between alfalfa revegetation and sludge biochar amendment was detected first in this study, suggesting the combination of these two variables improved soil physical properties was more intense than either in isolation, which was in accordance with other previous study [58].

#### 3.1.2. Effect on Soil Chemical Properties

**Figure 2:**
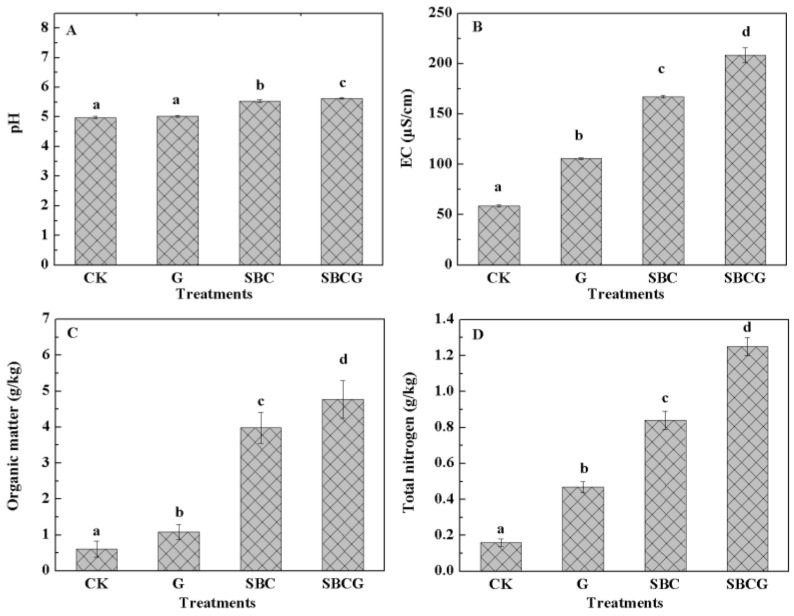

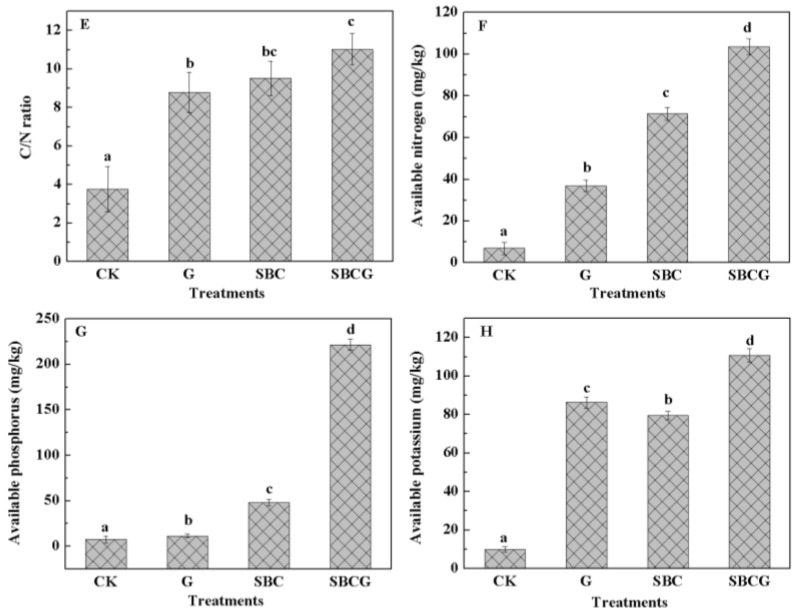
The chemical properties ((**A**–**H**) stand for pH, electrical conductivity, organic matter, total nitrogen, C/N ratio, available nitrogen, available phosphorus, available potassium, respectively) of IAC mining wasteland soil in the un-amended control (CK), alfalfa revegetation (G), sludge biochar amendment (SBC), and combined amendment (SBCG) treatments. Different characters, a, b, c, and d were used to indicate significant differences at *p* < 0.05. Vertical bars indicate standard deviation.

The soil chemical properties of the four treatments, including soil pH, electrical conductivity, organic matter, total nitrogen, C/N, available nitrogen, available phosphorus and available potassium, are shown in Figure 2. The SBC and SBCG treatments had significantly higher pH than CK (*p* < 0.05), indicating the significantly acidic decrease in soils from IAC mining wastelands for sludge biochar application (*p* < 0.05).

The effect of sludge biochar on soil pH was attributed to the relatively high pH (6.17 ± 0.03), which is higher than that of municipal sludge (5.64 ± 0.18) but lower than in most biochars (>8.0) [59,60]. In the slow pyrolysis process, many cations (such as Ca^2+^, Mg^2+^, K^+^ and Na^+^) form carbonates or oxides, and can reduce soil acidity [61] when sludge biochar is applied to soil, which is consistent with the effects of biochar on strongly acidic soils [62,63]. EC, organic matter, total nitrogen, available nitrogen, and available phosphorus in treated soil (G, SBC, and SBCG) increased significantly, by 0.8–2.6 times, 78.6%–6.82 times, 1.9–6.8 times, 4.3–13.8 times, and 0.5–27.8 times, respectively, over CK values, and the highest values were in the SBCG treatment. The difference in C/N ratio between SBCG (11.03 ± 0.81) and CK (3.77 ± 1.17) was significant (*p* < 0.05) but that between G and SBC was not (*p* > 0.05). Available potassium increased from 9.91 ± 0.42 in CK to 86.30 ± 0.86, 79.55 ± 1.17 and 110.79 ± 0.44 in the G, SBC, and SBCG treatments, respectively. This suggested that alfalfa revegetation and sludge biochar amendment significantly increased the contents of soil organic matter, nutrients and their availability (available nitrogen, available phosphorus and available potassium levels) (*p* < 0.05). Our results are consistent with the effects of biochar use and alfalfa revegetation on soil chemical properties from other reports. Many studies have demonstrated that the use of biochar can significantly increase soil organic C [64,65], mineral nutrient content (e.g., N, P, K) [66] and the cation exchange capacity of soil [18] for its carbon-rich and porous structure [56] and huge specific surface area [67]. Increasing quantities of data have shown that alfalfa revegetation can significantly increase soil soil organic carbon and total nitrogen concentrations present in the root ball [68], C/N ratio [57], porosity, aeration conditions, and water-holding capacity [49], all of which could accelerate the mineralization of organic carbon and the release of mineral nutrients from sludge biochar, and in turn promote the alfalfa growth. In the present study, an additive effect on soil chemical properties, like that of soil physical properties, was also detected when combined sludge biochar amendment and alfalfa revegetation (Figure 2).

### 3.2. Response of Soil Microbial Community to Alfalfa Revegetation and Sludge Biochar Amendment

#### 3.2.1. Soil Microbial Alpha Diversity

After de-multiplexing and quality filtering, 468,000 and 360,000 high-quality bacterial and fungal sequences for each treatment were obtained for further analysis, and the results were shown in Table 3.

The mean length of bacterial and fungal sequences of all samples was 229.99 and 208.67 bp, respectively. The number of bacterial and fungal OTUs as well as ACE, Chao1 and Shannon indices among the four treatments were CK < SBC < G < SBCG, whereas the Simpson index showed an opposite trend: CK > SBC > G > SBCG. This indicated that alfalfa revegetation and sludge biochar amendment significantly increased soil microbial diversity in IAC mining wastelands (*p* < 0.05), with an additive effect generated by their combined amendment. The results were in accordance with previous studies in biochar-enriched Terra preta soils [69,70] and alfalfa amended soils [71,72]. This increased microbial diversity might be due to the supply of a more comfortable habitats, many nutrients or mineralized organic matter from sludge biochar [6,73] and root exudates of alfalfa [74]. Across all soil samples, the gene coverage was more than 0.98, indicating that the sequencing results were representative.

#### 3.2.2. Soil Microbial Community Structure

**Figure 3:**
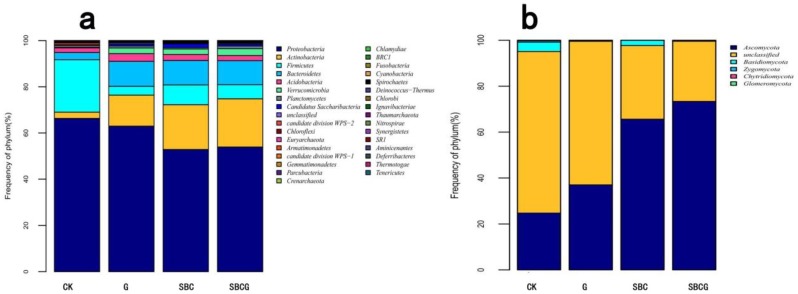
Frequency of bacterial (**a**) and fungal (**b**) phyla detected in IAC mining wasteland soil in the un-amended control (CK), alfalfa revegetation (G), sludge biochar amendment (SBC), and combined amendment (SBCG) treatments. Abundance is presented in terms of the average percentage, classified by Ribosomal Database Project (RDP) classifier at a confidence threshold of 80%. “Other” refers to the sum of unclassified sequences and all other taxa with abundances < 0.9% in any sample.

We detected 5927 OTUs in bacterial communities across all four treatments, for a total of 24 phyla, 48 classes, 97 orders, 236 families, and 737 genera. As shown in Figure 3a, the bacterial communities at the phylum level varied among the four treatments. The relative abundances of dominant bacteria ranked as follows: *Proteobacteria* > *Actinobacteria* > *Firmicutes* > *Bacteroidetes* > *Acidobacteria* > *Verrucomicrobia* > *Planctomycetes*. These seven phyla accounted for more than 97% gene sequences of soil in each treatment, with the respective proportion of 58.96%, 14.10%, 10.31%, 8.73%, 2.53%, 2.03%, and 0.88% in total. Alfalfa revegetation (G), sludge biochar amendment (SBC), and the combined amendment (SBCG) significantly reduced the relative abundances of *Proteobacteria*, and *Firmicutes* in soil of IAC mining wastelands (*p* < 0.05), whereas significantly increased abundances of *Actinobacteria*, *Bacteroidetes*, and *Verrucomicrobia* (*p* < 0.05).

The results were in agreement with other reports showing that *Proteobacteria*, *Actinobacteria* and *Firmicutes* were the main phyla in biochar [75,76,77] and alfalfa [78,79] amended soil, with the relative abundance of *Proteobacteria* decreased in biochar treated soil [77]. In addition, *Actinobacteria* was demonstrated to be the representative specie in recalcitrant carbon-rich soils like Terra preta [70] and pyrogenic carbon-treated soils [80], which explained the increased abundance of *Actinobacteria* in our study.

We detected 2974 OTUs in fungal community across all four treatments, for a total of 6 phyla, 24 classes, 72 orders, 154 families, 280 genera, and 368 species. The relative abundance of fungal community at the phylum level varied among the four treatments (Figure 3b). The relative abundances of dominant fungi ranked as follows: *Ascomycota* > *Basidiomycota* > *Zygomycota* > *Chytridiomycota* > *Glomeromycota.* The five phyla accounted for more than 50% gene sequences of soil in each treatment, with the respective proportion of 50.14%, 1.72%, 0.22%, 0.05% and 0.005% in total.

**Figure 4:**
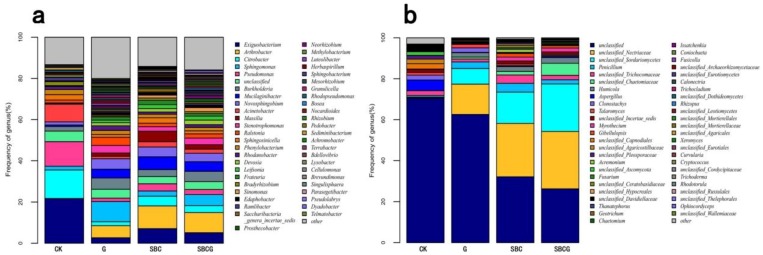
Frequency of bacterial (**a**) and fungal (**b**) genera detected in IAC mining wasteland soil in the un-amended control (CK), alfalfa revegetation (G), sludge biochar amendment (SBC), and combined amendment (SBCG) treatments. Abundance is presented in terms of the average percentage, classified by RDP classifier at a confidence threshold of 80%. “Other” refers to the sum of unclassified sequences and all other taxa with abundances < 0.9% in any sample.

The results were quite similar to the findings in a previous study [81], which showed that *Ascomycota*, *Basidiomycota*, and *Zygomycota* were the dominant fungal phyla across all soil samples while *Chytridiomycota* and *Glomeromycota* were minor phyla. Alfalfa revegetation (G), sludge biochar amendment (SBC), and the combined amendment (SBCG) significantly elevated the relative abundance of *Ascomycota* in the soil of IAC mining wastelands (*p* < 0.05), and significantly reduced that of *Basidiomycota* (*p* < 0.05). At the genus level, there was a total of 737 bacterial genera (Figure 4a) detected in four treatments, with different patterns of dominance. Alfalfa revegetation, sludge biochar amendment, and the combined amendment significantly elevated the relative abundances of *Arthrobacter, Burkholderia. Devosia, Edaphobacter, Leifsonia, Massilia, Mucilaginibacter, Sinomonas, Sphingomonas* and *Stenotrophomonas* in soil (*p* < 0.05), whereas significantly reduced abundances of *Exiguobacterium*, *Citrobacter*, *Pseudomonas,* and *Bradyhizobium* (*p* < 0.05). *Arthrobacter,* an gram-negative bacterium, was demonstrated to have the ability to degrade hydrocarbons and its’ higher relative abundance was detected in biochar [82,83] and alfalfa-amended soil [84]. *Sphingobium* was reported to increase in soil treated with biochar, and can degrade recalcitrant compounds [85]. A total of 280 fungal genera, including 368 species, were detected across four treatments, with different patterns of dominance (Figure 4b). Alfalfa revegetation, sludge biochar amendment, and the combined amendment significantly enhanced the relative abundances of unclassified*_Nectriaceae*, unclassified*_Sordariomycetes*, *Penicillium*, *Humicola,* unclassified*_Chaetomiaceae*, and *Myrothecium* (*p* < 0.05), whereas significantly decreased that of *Aspergillus*, unclassified*_Capnodiales*, unclassified*_Agaricostilbaceae*, *Clonostachys*, unclassified_*Ascomycota*, unclassified_*Pleosporaceae,* and unclassified_*Davidiellaceae* (*p* < 0.05). Our results were in accord with the relative abundance change of fungal community in biochar [76,86,87] and alfalfa [79,88] amended soil.

**Figure 5:**
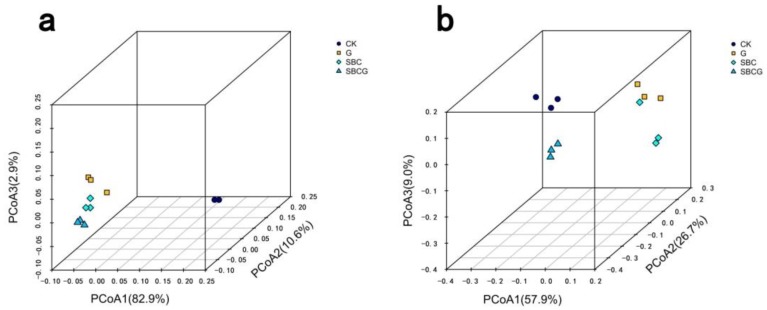
Principal coordinate analysis of bacterial (**a**) and fungal (**b**) genera in IAC mining wasteland soil in the un-amended control (CK), alfalfa revegetation (G), sludge biochar amendment (SBC), and combined amendment (SBCG) treatments.

**Figure 6:**
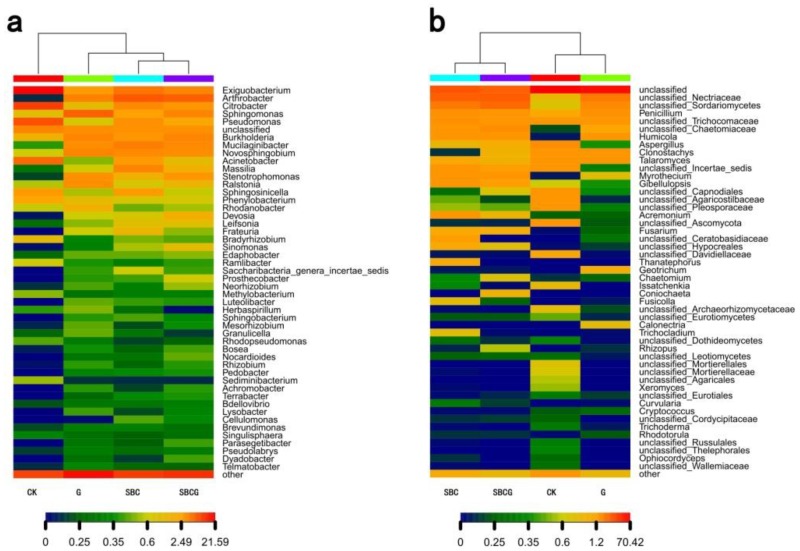
Heat-map based on bacterial (**a**) and fungal (**b**) community composition (at the genus level) in IAC mining wasteland soil in the un-amended control (CK), alfalfa revegetation (G), sludge biochar amendment (SBC), and combined amendment (SBCG) treatments.

PCoA (Figure 5) and hierarchical cluster heat-map analysis (Figure 6) revealed the similarity and differences of bacterial and fungal communities in the four treatments at the genus level. The results of PCoA showed that PCoA 1, PCoA 2, and PCoA 3 explained 82.9%, 10.6%, and 2.9%, respectively, as well as 57.9%, 26.7%, and 9%, respectively, differences of bacterial (Figure 5a) and fungal (Figure 5b) community structure in soil. Heat-map analysis suggested that for bacterial communities (Figure 6a), CK treatment alone formed its own cluster and the three amendment treatments (G, SBC, and SBCG) formed a separate cluster, indicating the similarity of amendment treatments (G, SBC, and SBCG) and the differences between amended (G, SBC, and SBCG) and un-amended (CK) treatments. For fungal communities (Figure 6b), CK and G were clustered together while SBC and SBCG were clustered together, indicating that sludge biochar amendment had a greater effect on soil fungal community than alfalfa revegetation.

Our results suggested the significant change of bacterial and fungal communities’ compositions for sludge biochar application and alfalfa revegetation, which were in agreement with previous studies. Organic amendments were demonstrated to be the most important means of managing soil biodiversity, and their quantity, quality, and distribution each affected the trophic structure of the soil food web [89,90]. Sludge biochar and root exudates of alfalfa both provided organic matter for soil microbes’ growth. Generally, the amount of soil organic matter from sludge biochar was larger than that from root exudates of alfalfa, which were in agreement with the much higher organic matter value of SBC than of G (Figure 2). Additionally, fungi were known to be saprophytes, associated with degradable soil organic matter [91]. Normally, soil organic matter was successively utilized by bacteria and fungi. All the aforementioned could explain the bacterial difference between control (CK) and treated soils (G, SBC, SBCG) as well as the fungal difference between sludge biochar amended (SBC and SBCG) and un-amended soils (CK and G).

### 3.3. The Complex Relationship between Soil Physicochemical Properties and Microbial Communities, and the Response of Plant Growth to Remediation

#### 3.3.1. Redundancy Analyses of Soil Physicochemical Properties and Microbial Community

**Figure 7:**
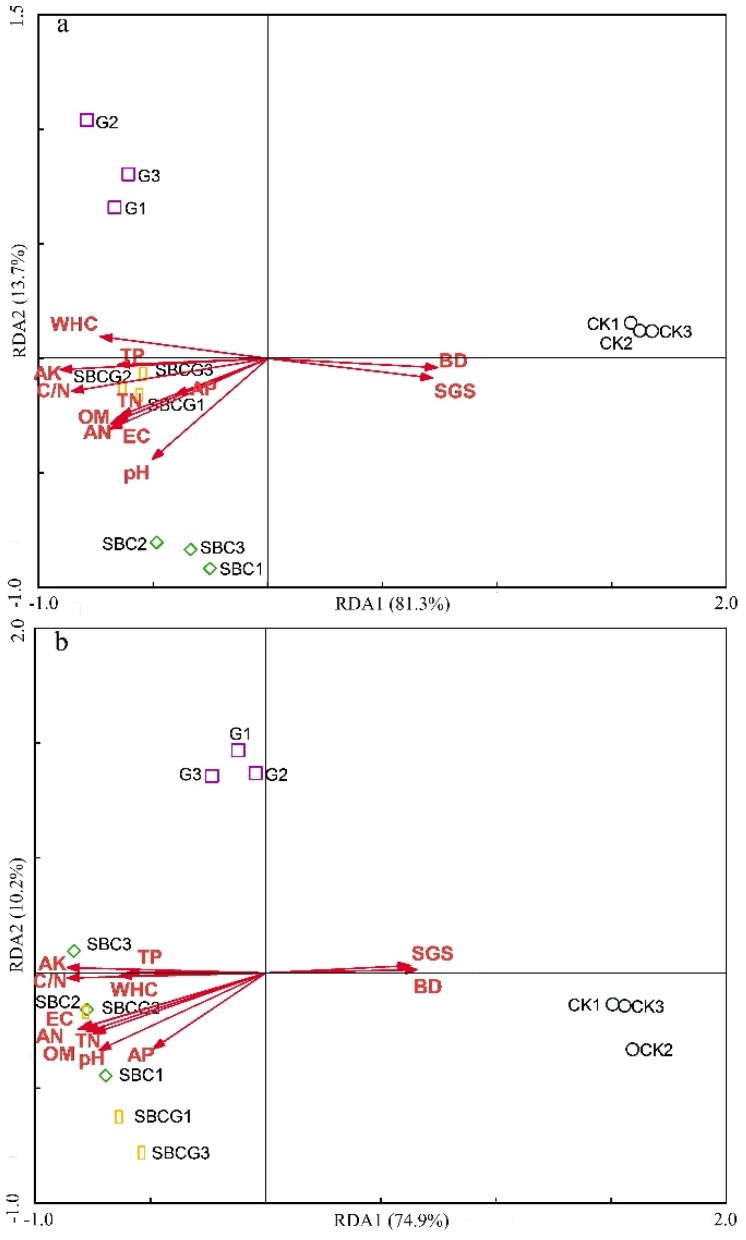
Redundancy analysis (RDA) between soil bacterial communities (**a**) or fungal communities (**b**) and environmental factors in the four different treatments: un-amended control (CK), alfalfa revegetation (G), sludge biochar amendment (SBC), and combined amendment (SBCG) treatments.

Redundancy analyses between soil properties and bacterial and fungal community structure were conducted and a bi-plot is shown in Figure 7. The investigated soil physicochemical properties could explain 98.9% and 99.0% of the variation of bacterial and fungal community structure (Table 4), respectively. All soil physicochemical properties (except available phosphorus) had a significant impact on bacterial community structure (*p* < 0.05). The effects of available potassium, pH, C/N, bulk density, water-holding capacity, specific gravity, EC, total nitrogen, and total porosity were significant (*p* < 0.01). All soil physicochemical properties had a significant influence on fungal community (*p* < 0.05). The effects of pH, available potassium, C/N, EC, available nitrogen, total nitrogen, organic matter, and bulk density were significant (*p* < 0.01). The variation in the bacterial community explained by the soil physicochemical properties decreased as follows: available potassium > pH > C/N > bulk density > water-holding capacity > specific gravity > EC > available nitrogen > total nitrogen > organic matter > total porosity > available phosphorus. Furthermore, available potassium, pH, C/N, organic matter, bulk density, and total porosity could be used to explain 93.6% variation of the bacterial community data, and were confirmed to be the key environmental factors. The variation in fungal community explained by soil physicochemical properties decreased as follows: pH >available potassium > C/N > EC > available nitrogen > total nitrogen > organic matter > bulk density > water-holding capacity > specific gravity > total porosity > available phosphorus. Moreover, available potassium, pH, organic matter, C/N, bulk density, and total porosity could be used to explain 88.0% variation of fungal community data, and were confirmed to be the critical environmental factors. In general, the key environmental factors affecting bacterial community were similar to those affecting the fungal community.

Our results suggested that alfalfa revegetation could enhance soil microbial community diversity and richness of IAC mining wastelands, which are similar to the results of Chen [92]. Alfalfa revegetation significantly improved soil physicochemical properties, including enhancement of soil porosity, water-holding capacity and content of organic matter and nutrients (e.g., N, P, and K), decrease of soil bulk density, and amelioration of soil structure, all of which were helpful to the proliferation of soil microorganisms (bacteria and fungi). Sludge biochar amendment also enhanced the diversity and richness of the microbial community in IAC mining wasteland soil. This is because biochar not only provides several types of nutrients (C, N, and other trace elements) for soil microbial growth [93], but will also ameliorate the soil environment for microbial proliferation, by for example reducing soil acidity, elevating soil porosity, and improving soil aeration conditions [94]. These claims are consistent with the results of biochar on microbiota in acidic soil [76,95]. Additionally, due to its porous structure, large specific surface area and cation exchange capacity, biochar has the ability to retain nutrients [96] and would provide them to the soil after oxidation. The combination of alfalfa revegetation and sludge biochar amendment further enhanced the diversity and richness of the soil microbial community in IAC mining wastelands. This is because sludge biochar not only directly increases soil microbial diversity and richness, but also benefits alfalfa growth to affect soil microbiota. The aromatic hydrocarbon structure contributes to the long-term retention of sludge biochar in soils [18], and thus provides nutrients continuously to soil after its oxidation.

#### 3.3.2. Responses of Plant Growth and Root Morphology

**Figure 8:**
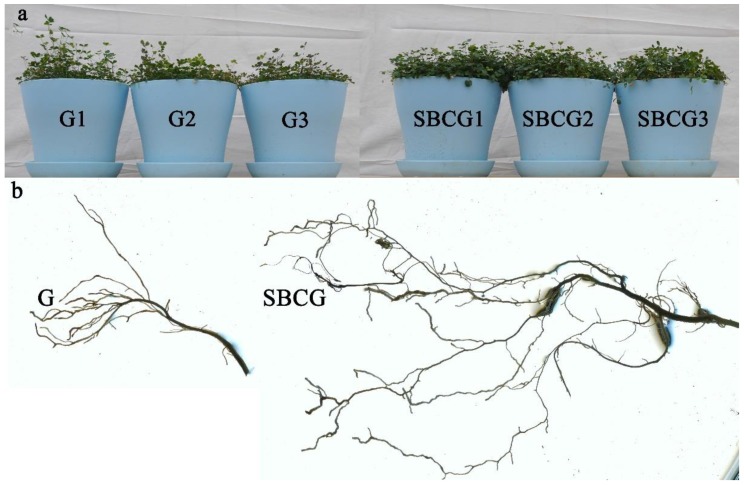
Alfalfa growth (**a**) and root morphology (**b**) in the alfalfa revegetation (G) and combined alfalfa and sludge biochar amendment (SBCG) treatments.

Alfalfa growth (Figure 8a) and its root morphology (Figure 8b) in the G and SBCG treatments differed. Alfalfa growth in the sludge biochar amendment treatment (SBCG) was significantly better than the alfalfa-only treatment (G) (Figure 8a). Plant height, shoot biomass, root biomass, and total biomass were significantly higher in SBCG than in G (Table 5). The root segment of alfalfa had similar results, and TRL, RSA, RV (root volume, see Part 2.5), RAD, RTN, and RFN of SBCG were significantly larger than those of G (Table 6, *p* < 0.05).

Sludge biochar application could continuously ameliorate the soil environment (physicochemical properties) of IAC mining wastelands. It may reduce soil acidity, specific gravity, and bulk density, improve soil texture and ventilation conditions, enhance soil porosity, water-holding capacity, the content and effectiveness of nutrients, and change the microbial community structure to promote the growth of alfalfa [97,98].

## 4. Conclusions

Alfalfa revegetation and sludge biochar amendment both improved soil physicochemical properties and enhanced the diversity and richness of the microbial community. In addition, the combined treatment (soil amended with alfalfa revegetation and biochar) resulted in the greatest improvement of soil physicochemical properties, the enhancement of diversity and richness of microbial community, and the promotion of plant growth. Redundancy analyses showed that soil physicochemical properties could explain 98.9% and 99% of the variation in bacterial and fungal community structure, respectively, and soil available potassium, pH, organic matter, C/N ratio, bulk density, and total porosity were the critical environmental factors affecting soil microbiota. Moreover, sludge biochar could be used to promote the growth of alfalfa and change their root morphology, which in turn accelerated the soil rehabilitation process of IAC mining wastelands. In this way, the combined amendment of alfalfa revegetation and sludge biochar amendment not only serve as soil remediation for IAC mining wastelands but also resolve the difficult problem of municipal sludge disposal by making the waste profitable. Thus, a combined strategy is recommended to achieve sustainable soil restoration for IAC mining wastelands.

## Figures and Tables

**Table 1:** Properties of soil and sludge biochar used in experiments.

Samples	Soil	Sludge Biochar
Clay (%)	7.64 ± 0.27	-
Silt (%)	15.48 ± 0.32	-
Sand (%)	76.88 ± 0.38	-
pH	5.44 ± 0.02	6.17 ± 0.03
Electrical conductivity (μS/cm)	45.16 ± 1.03	272.67 ± 6.06
Organic carbon (g/kg)	0.73 ± 0.18	97.32 ± 2.78
Total nitrogen (g/kg)	0.21 ± 0.03	2.48 ± 0.02
C/N ratio	3.44 ± 0.37	39.31 ± 0.72
Available nitrogen (mg/kg)	10.35 ± 2.58	378.93 ± 10.19
Available phosphorus (mg/kg)	9.43 ± 2.31	712.61 ± 12.24
Available potassium (mg/kg)	10.62 ± 1.51	172.97 ± 5.92
BET surface area (m^2^/g)	-	47.03 ± 0.02
Pore volume (cm^3^/g)	-	0.06 ± 0.001
Pore size (nm)	-	5.16 ± 0.01

**Table 2:** Four treatments were designed in this study.

Treatments	Mine Soil (kg)	Alfalfa Seed (g)	Sludge Biochar (kg)
CK	5	-	-
G	5	5	-
SBC	4.75	-	0.25
SBCG	4.75	5	0.25

Notes: CK, the control soil; G, soil planted with alfalfa; SBC, soil amended with sludge biochar; SBCG, soil amended with sludge biochar and planted with alfalfa.

**Table 3:** Summary of the number of bacterial and fungal sequences, number of operational taxonomic units (OTUs), α-diversity indices (based on 97% OTUs) and coverage in four treatments (mean ± S. V. (standard deviation), *n* = 3).

Microbial Group	Indices	CK	G	SBC	SBCG
Bacteria	Number of OTUs	927 ± 36 a	1332 ± 15 b	1278 ± 62 b	1341 ± 8 b
	ACE	3220 ± 395 a	3366 ± 192 a	3270 ± 221 a	3430 ± 157 a
	Chao 1	2309 ± 240 a	2603 ± 166 a	2565 ± 142 a	2630 ± 84 a
	Shannon	3.55 ± 0.04 a	4.89 ± 0.02 c	4.62 ± 0.02 b	4.98 ± 0.07 c
	Simpson	0.092 ± 0.00 d	0.020 ± 0.00 c	0.028 ± 0.00 c	0.017 ± 0.00 b
	Coverage	0.99 ± 0.00 a	0.98 ± 0.00 b	0.98 ± 0.00 a	0.98 ± 0.00 a
Fungi	Number of OTUs	361 ± 17 a	496 ± 7 a	427 ± 18 a	514 ± 91 a
	ACE	1074 ± 107 a	1558 ± 27 b	1085 ± 89 a	1798 ± 69 b
	Chao 1	752 ± 42 a	1093 ± 111 b	1079 ± 131 b	1157 ± 52 b
	Shannon	1.96 ± 0.07 a	3.07 ± 0.02 b,c	2.72 ± 0.07 b	3.32 ± 0.02 c
	Simpson	0.340 ± 0.02 b	0.107 ± 0.00 a	0.140 ± 0.00 a	0.011 ± 0.00 a
	Coverage	0.99 ± 0.00 a	0.99 ± 0.00 a	0.99 ± 0.00 a	0.98 ± 0.00 a

Note: Different letters (such as a, b, c, d) within a row indicate significant differences (*p* < 0.05).

**Table 4:** Results of the Monte Carlo permutation test for bacterial and fungal community variation explained by soil physicochemical properties.

Soil Parameters	Bacterial Variation Explains (%)	*F*-Value	*p*-Value	Soil Parameters	Fungal Variation Explains (%)	*F*-Value	*p*-Value
Available potassium	74.3	28.955	0.0020	pH	72.5	26.356	0.0020
pH	67.1	20.375	0.0080	Available potassium	64.9	18.496	0.0020
C/N	67.0	20.316	0.0040	C/N	64.6	18.283	0.0020
Bulk density	49.9	9.950	0.0040	EC (Electrical conductivity)	59.5	14.677	0.0020
Water-holding capacity	49.4	9.754	0.0040	Available nitrogen	57.9	13.765	0.0020
Specific gravity	47.5	9.061	0.0060	Total nitrogen	54.8	12.128	0.0020
EC (Electrical conductivity)	46.7	8.769	0.0040	Organic matter	51.2	10.491	0.0020
Available nitrogen	46.1	8.555	0.0120	Bulk density	39.3	6.488	0.0240
Total nitrogen	43.1	7.575	0.0100	Water-holding capacity	37.2	5.930	0.0180
Organic matter	40.8	6.879	0.0120	Specific gravity	36.7	5.790	0.0220
Total porosity	39.2	6.456	0.0040	Total porosity	32.3	4.761	0.0440
Available phosphorus	17.2	2.074	0.1420	Available phosphorus	26.0	3.522	0.0420
Total	98.9			Total	99.0		

**Table 5:** The plant height, root length, shoot biomass, root biomass, and total biomass of alfalfa after incubation (mean ± S.V. (standard deviation), *n* = 3).

Treatments	Plant Height (cm)	Shoot Biomass (g)	Root Biomass (g)	Total Biomass (g)
G	8.98 ± 0.24 a	5.36 ± 0.35 a	8.53 ± 0.03 a	13.89 ± 0.34 a
SBCG	11.09 ± 0.15 b	7.64 ± 0.26 b	11.55 ± 0.04 b	19.20 ± 0.22 b

Different letters within each column indicate significant differences (*p* < 0.05). G, soil planted with alfalfa; SBCG, soil amended with 5% (*w*/*w*) sludge biochar and planted with alfalfa. Shoot, root and total biomass of alfalfa were dry weight biomass as stated in Section 2.5.

**Table 6:** Summary of root growth indices of alfalfa after incubation (mean ± S.V. (standard deviation), *n* = 3).

Treatments	TRL (cm)	RSA (cm^2^)	RV (cm^3^)	RAD (mm)	RTN	RFN
G	96.70 ± 1.79 a	12.05 ± 1.17 a	0.19 ± 0.04 a	0.46 ± 0.02 a	161.67 ± 5.36 a	293.00 ± 6.56 a
SBCG	120.67 ± 4.35 b	18.38 ± 0.96 b	0.53 ± 0.04 b	0.58 ± 0.01 b	249.00 ± 5.69 b	365.00 ± 18.90 b

Different letters within each column indicate significant differences (*p* < 0.05). G, soil planted with alfalfa; SBCG, soil amended with 5% (*w*/*w*) sludge biochar and planted with alfalfa. TRL, total root length; RSA, root surface area; RV, root volume; RAD, root average diameter; RTN, root tip number; RFN, root fork number.
